# Scavenger receptor class A, member 5 is associated with thyroid cancer cell lines progression via epithelial‐mesenchymal transition

**DOI:** 10.1002/cbf.3455

**Published:** 2020-01-27

**Authors:** Chen Zheng, Er‐Jie Xia, Rui‐Da Quan, Adheesh Bhandari, Ou‐Chen Wang, Ru‐Tian Hao

**Affiliations:** ^1^ Department of Thyroid and Breast Surgery The First Affiliated Hospital of Wenzhou Medical University Wenzhou Zhejiang People's Republic of China

**Keywords:** SCARA5, PTC, EMT

## Abstract

Thyroid cancer (TC) has become one of most common endocrine malignancies in recent decades. Due to gene background polymorphism, it's outcome goes quite differently in each patient. For exploring the mechanism, we performed whole transcriptome sequencing of paired papillary thyroid carcinoma (PTC) and adjacent thyroid tissues. As a result, scavenger receptor class A member 5 (SCARA5) might be a crucial anti‐oncogene associated with PTC. By RT‐qPCR, we first detected the expression of SCARA5 in PTC tissue and three type of TC cell lines. Besides, The Cancer Genome Atlas (TCGA) data were gathered to analysis the relationship between SCARA5 and clinical feature. A series of loss‐function experiments in TC cell lines (KTC‐1 and BCPAP) to investigate the function of SCARA5 in PTC. The results showed that SCARA5 expression in PTC was lower than adjacent normal tissue. And, it's consistent with the TCGA database. After analyse the correlation between SCARA5 expression and clinicopathological features in TCGA database, we discovered that downregulated SCARA5 is significantly connected age (*P* = .04) and tumour size (*P* = .032). Knockdown of SCARA5 in TC cell line could significantly increase the function of cells proliferation, colony formation, migration, and invasion. Furthermore, we also proved that SCARA5 could modulate the expression of epithelial‐mesenchymal transition‐related proteins, which influence invasion and migration. To best of our knowledge, SCARA5 is a suppressor gene which was associated with PTC and might be a potential therapeutic target in the future.

**Significance of the study:**

Thyroid cancer (TC) has become one of most common endocrine malignancies in recent decades. By whole transcriptome sequencing of paired papillary thyroid carcinoma (PTC) and adjacent thyroid tissues, author discovered that scavenger receptor class A member 5 (SCARA5) might be crucial anti‐oncogene associated with PTC. Furthermore, knocking‐down of SCARA5 in TC cell line can increase the function of cells proliferation, colony formation, migration, and invasion. Author also proved that SCARA5 could modulate the expression of epithelial‐mesenchymal transition‐related proteins.

## 1 INTRODUCTION

Thyroid cancer (TC) has become one of most frequent malignancies of the endocrine system in recent decades.^1^ The U.S. National Cancer Institute anticipated 53 990 new cases besides 2110 deaths numbers of patients due to TC in the USA in 2018.^2^ A considerable study also pointed out that global TC cases has increased 4% approximately in each year, and predicted it in 2030 will surpass colorectal cancer become as the fourth most commonly diagnosed cancer.^3^ TC is generally classified into four different histological types, including papillary thyroid carcinoma (PTC), follicular thyroid carcinoma (FTC), anaplastic thyroid carcinoma, and medullary thyroid carcinoma.^4^ Among those subtypes, PTC nearly accounts for 80% to 85% of all TC cases.^5^

Due to patient individual gene background, different PTC patient suffer complete different clinical outcomes.^6^ Accumulating studies had published that the genomic mutations such as stimulation of oncogene or silencing of tumour suppressor genes is a crucial step in the development and tumorigenesis of TC.^7^, ^8^, ^9^ In particular, B‐type Raf kinase V600E, a famous gene mutation, can accelerate PTC tumorigenesis and progression via abnormally triggering the mitogen‐activated pathway kinase pathway.^10^ Besides, another notable mutations such as RAS mutation,^11^ TERT mutation,^12^ PTEN mutation,^13^, ^14^ PIK3CA mutation^15^, ^16^ and TP53 mutation^17^, ^18^ also play a significant role in thyroid carcinoma. Whereas numerous scholars had made remarkable progress in TC research in decades, but there are still many underlying molecular mechanisms yet remain unclear.

Scavanger receptors (SRs) are a type of receptor superfamily which contains one or several repeats of highly conserved SRCR domain protein module.^19^ Many biomolecules such as modified lipoproteins, lipids, polyribonucleotide and polysaccharides are able to combine this membrane receptor.^20^ In the previous study, SRs had been divided into eight discrete classes based on their ability to identify different type of low density lipoprotein.^21^ In 2006, SCARA5 had been discovered and identified as the new member of this superfamily.^22^ Unlike the other family members mainly detected in macrophages, SCARA5 was widely expressed in diverse human tissues such as bladder, ovary, kidney, testis, adrenal gland, skin and trachea.^23^, ^24^

Recently, an investigator have defined function of SCARA5 was that it can serve as nontransferrin iron delivery receptor to bound iron and help to the developing kidney.^25^

More intriguingly, there are accumulating studies validated that SCARA5 act as a tumour suppressor in many human cancer. In human hepatocellular carcinoma (HCC), Huang et al found that SCARA5 expression have been down‐regulated because of promoter hypermethylation and loss of heterozygosity; suppression of SCARA5 was also correlates with cellular invasion and overall progression.^26^ A considerable report noted that up‐regulation of SCARA5 could inhibit lung cancer lines tumorigenesis and progression in vitro.^27^ Furthermore, Ying Liu et al testified that inhibition of SCARA5 could promote tumorigenicity, colony formation, cell invasion and metastasis in oral squamous cell carcinoma.^28^ Emerging shreds of evidence have manifested that SCARA5 act as effective anti‐tumour gene in many tumour, however, whether the SCARA5 gene have essential role in PTC still remains unclear.

With the help of sequencing technology, we have been revealed whole transcriptome bioinformation of 19 pairs PTC samples and its adjacent normal thyroid tissues.^29^ The results showed that SCARA5 might act as a vital anti‐tumour gene in the PTC. By RT‐qPCR, we performed 57 pairs PTC samples to further validated this sequencing result. Besides, the connection between SCARA5 expression and the clinicopathologic characteristics was analysed in The Cancer Genome Atlas (TCGA) cohort. Loss of function experiments was conducted in two TC cell lines (KTC‐1 and BCPAP) via using small interfering RNA (Si‐RNA). To best our knowledge, SCARA5 is a suppressor gene which was associated with PTC and might be a potential biomarker in the future.

## 2 MATERIALS AND METHODS

### 2.1 Patients and tissue collection

A total of 57 pairs of matched primary PTCs and matched noncancerous tissues were selected for validated cohort after patient initial surgery. All enrolled patient were performed surgery at the First Affiliated Hospital of Wenzhou Medical University between 2014 and 2018. None of patients acquired chemo‐therapy or radio‐therapy for pre‐treatment. All samples were instantly obtained at the time of lesion resection and were frozen in liquid nitrogen immediately. To ensure the quality of the RNA, we stored all samples at −80°C refrigerator before RNA extraction. Furthermore, for ensure determine histologic diagnosis, all tumour tissues were retrospectively examined by two senior pathologists. Ethical approval for this study was obtained from the Ethics Committee of The First Affiliated Hospital of Wenzhou Medical University. Patients signed informed consent forms, and research protocols for the use of tissues were approved by and conducted in accordance with the ethical standards of the Institutional Review Board of The First Affiliated Hospital of Wenzhou Medical University (approval no. 2012‐57).

### 2.2 The Cancer Genome Atlas database

TC RNA‐seq data and corresponding clinical information were downloaded from the TCGA database (https://tcgadata.nci.nih.gov/tcga/). In result, total of 502 TCs with complete clinicopathologic features such as age, gender, lymph node metastasis, tumour size, clinical stage (ACJJ7), and histological type were collected.

### 2.3 RNA extraction and RT‐qPCR

For RNA isolation, TRIzol reagent (Invitrogen, Thermo Fisher Scientific, Inc.) was used to isolate 60 pairs of tissue specimens and TC cell line samples according to manufacturer's protocol. The A260/A280 ratio and spectrophotometric value were used to assess the RNA quality and quantity, respectively. As for cDNA synthesizing, we were used ReverTra Ace qPCR RT Kit (Toyobo, Osaka, Japan) at 16°C for 5 minutes, 42°C for 30 minutes and 98°C for 5 minutes. Real‐time PCR analysis was performed in triplicate on an ABI Prism 7500 sequence detection system (Thermo Fisher Scientific, Inc.) by using the THUNDERBIRD SYBR qPCR Mix (TOYOBO) following the manufacturer's protocol. The thermocycling conditions were: 95°C for 2 minutes, followed by 40 cycles of 95°C for 15 seconds and 60°C for 60 seconds, and a final step of 72°C for 5 minutes. The relative expression of mRNA was calculated using the 2^−ΔΔCq^ method.^30^ The primer sequences for PCR are as follows: SCARA5, forward 5′‐CAGCTGGTTTCTTACCACGTAT‐3′ and reverse 5′‐GCACAAGTTCTCCCACACTTAG‐3′; GAPDH, forward 5′‐GGTCGGAGTCAACGGATTTG‐3′ and reverse 5′‐ATGAGCCCCAGCCTTCTCCAT‐3′.

### 2.4 Cell lines and growth conditions

The TC cell line (TPC‐1, KTC‐1 and BCPAP) and normal thyroid (HTORI3) cell lines were purchased from the Chinese Academy of Sciences Stem Cell Bank. For growth conditions, above cell lines were cultured in RPMI 1640 (Invitrogen; Thermo Fisher Scientific, Inc., Waltham, Massachusetts) which was supplemented with 10% fetal bovine serum (FBS; Invitrogen; Thermo Fisher Scientific, Inc.), 1× MEM nonessential amino acids, and 1× sodium pyruvate. All cell lines were grown in a humidified incubator at 37°C in 5% CO_2_/95% air.

Above mentioned cell lines were obtained the ethical approval from the Ethics Committee of The First Affiliated Hospital of Wenzhou Medical University.

### 2.5 RNA interference

The siRNA of SCARA5 and control siRNA (siNC) were synthesized at Gene Pharma (Shanghai, China). The sequences of the SCARA5 are as follows: SCARA5 (sense‐1:5′‐GCUCCAUCUGUGAGGAUUCdTdT‐3′; antisense‐1:5′‐GAAUCCUCAGAUGGAGdTdT‐3′; sense‐2:5′‐UGGGCAUGCGUGGGUUCAAdTdT‐3′; antisense‐2:5′‐UUGAACCCACGCAUGCCCAdTdT‐3′). In our experiment, we randomly added sequences without any target sequence tracking as negative control (Si‐NC). Briefly speaking, we plated cells into six‐well plates. After 24 hours, according to the manufacturer's guide, we used Lipo iMAX (Invitrogen, Grand Island, New York) to transfect our target Si‐RNA into plated cell. The concentration of siRNA as follows: 100 nM for BCPAP and 75 nM for KTC‐1. After 48 hours, we harvested transfected cells for subsequent RNA expression analysis. All loss of function experiments were performed in triplicate.

### 2.6 CCK‐8 proliferation assay

Firstly, BCPAP and KTC‐1 cells (about 1.5 × 10^3^ cells) were plated into 96‐well plates. After 24 hours, we use above methods to transfect cells with siRNA. Subsequently, when we observed cell adherence, we added 10 μL of CCK‐8 solution into each well and incubated at 37°C for 2 to 4 hours. All cell plates were continuously incubated for 5 days. Final, we measured the absorbance of each wells at 450 nm and drawn cell proliferation curves. All experiments were performed in triplicate.

### 2.7 Colony formation assay

At the beginning, BCPAP and KTC‐1 cells (about 1.5 × 10^3^ cells) were seeded into 96‐well plates and consecutively incubated for 8 to 14 days. When we observed 50~70 cell in one colony formation at inverted light microscope (Olympus Corporation, Japan), the magnification is ×10. Then we use 4% paraformaldehyde (Sigma) to fix cell with for 30 minutes. Subsequently, 0.01% crystal violet stained cell for 30 minutes. All assays were performed in triplicate.

### 2.8 Migration and invasion assays

We used transwell chambers (Corning Costar Corp., Cambridge, Massachusetts) for cell migration assays. About 3 × 10^5^ cells/0.3 mL for KTC‐1 cells (3.5 × 10^5^/0.3 mL cells for BCPAP cells) were plated into the upper chamber. Besides, we put 0.6 mL of medium supplemented with 20% FBS in lower chamber. After 24 hours, we use a cotton swab remove the cells those have not migrate to lower surface of the well. Then we used 4% paraformaldehyde and 0.4% crystal violet to stained lower surface. The image was captured in the microscope (magnification is ×40) for further analysis.

BioCoat Matrigel Invasion Chambers (Corning, New York) was use for invasion assays. The procedure was similar with above protocol described for the migration assay.

### 2.9 Protein extraction and western blot analysis

The samples were cleaved in RIPA lysis buffer (Beyotime, Shanghai, China). By using SDS‐PAGE on a 10% gel and electro transferred to PVDF membranes total proteins (20 μg) in the lysate were removed. Using 5% skim milk (BD, Difco Skim Milk, 232100) the membranes were blocked for 2 hours and then were probed with primary antibodies overnight at 4°C. After rinsing three times with triple buffered saline and Tween 20, the cells were incubated for 2 hours with secondary antibody at room temperature. The primary antibodies used included vimentin (Abcam), N‐cadherin (Abcam), E‐cadherin (Abcam), and β‐actin (Sigma). Goat anti‐rabbit HRP‐conjugated IgG (Abcam) and goat anti‐mouse HRP‐conjugated IgG (Abcam) as the secondary antibody. β‐actin served as an internal control.

### 2.10 Statistical analysis

Statistical data assessments were accomplished using SPSS 23.0 software (SPSS, Inc., Chicago, Illinois). For creating the graphs, we use GraphPad Prism version 6.01 (GraphPad Software, Inc., La Jolla, California). Data on normal distribution were expressed as the mean ± SD. Otherwise, the differences between Si‐RNA and Si‐NC groups were estimated by student *t*‐test (two‐tailed). As for the clinical feature, patients of PTC were divided into two parts based on the high and low expression level of SCARA5, with the median value as the cutting line. *P* < .05 was considered to indicate a statistically significant difference.

## 3 RESULTS

### 3.1 SCARA5 is significantly downregulated in PTC

**Figure 1:**
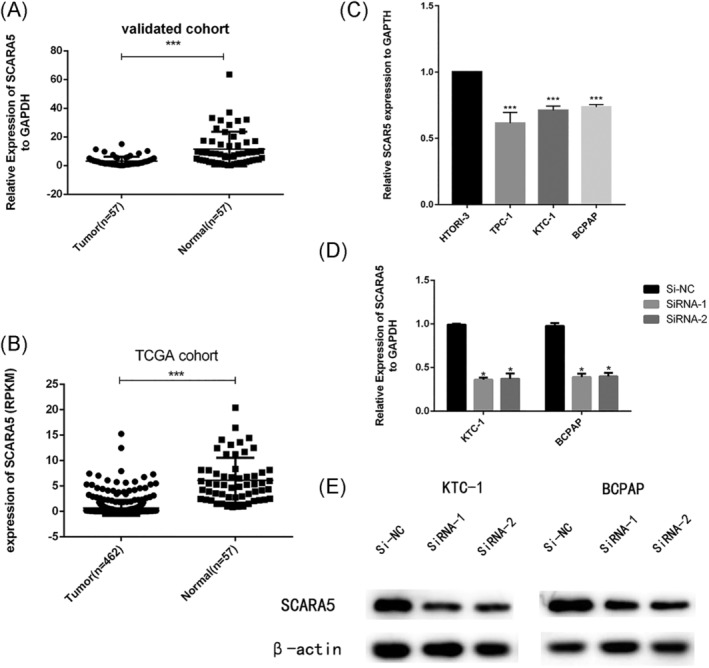
SCARA5 is underexpressed in human PTC tissues and cell lines. A, The expression of SCARA5 was significantly down‐regulated in the our cohort (*P* < .0001). B, The expression of SCARA5 was significantly down‐expressed in the TCGA cohort (*P* < .001). C, The relative expression of SCARA5 (compared with the GAPDH gene) was examined via RT‐qPCR. Compared to normal thyroid cell lines (HTORI‐3), TPC‐1, KTC‐1 and BCPAP cell lines have lower SCARA5 expression (*P* < .001). D, SCARA5 relative expression level (compared with the GAPDH gene) in KTC‐1 and BCPAP via RT‐qPCR. SCARA5 expression in Si‐RNA1 and Si‐RNA2 group was lower than corresponding Si‐NC group. E, The relative expression of SCARA5 (compared with the GAPDH gene) was verified by Western blotting in KTC‐1, BCPAP cell lines. Compared with the corresponding Si‐NC group, the expression of SCARA5 in Si‐RNA group was lower. *P < .05, **P < .01, ***P < .001 in comparison with Si‐NC or GAPDH using student's *t*‐test

In order to validate the results of whole transcriptome sequencing, we collected 57 paired of primary PTC tissue and its adjacent noncancerous tissue as validated cohort. Though RT‐qPCR, we noted that SCARA5 expression in normal thyroid tissue was significantly higher than PTC tissues (Figure 1A, *P* < .001). Whether SCARA5 is underexperssed in cell line, we also estimated the expression level of SCARA5 in three type TC cell line via RT‐qPCR. To further investigate the dysregulated expression of SCARA5, RNA sequencing data of TC was obtained from the TCGA database which contained with 462 cases of TC patients and 57 pairs of patients with normal tissue (Figure 1B). After analysing this results, we identified that SCARA5 act as an anti‐oncogene involved in PTC tumorigenesis.

### 3.2 SCARA5 expression was associated with clinicopathologic characteristics in PTC

To facilitate in‐depth investigation of SCARA5 in PTC, we found that many clinical features of PTC were correlated with expression of SCARA5 both in our validated cohort and TCGA cohort. Based on the median value, the TCGA cohort was divided into two parts which are low expression and high expression groups respectively. As showed in Table 1, result showed that SCARA5 expression was correlated with Age (*P* = .04), Tumour size (*P* = .032) and disease stage (*P* = .003) (based on the seventh edition of the American Joint Committee on Cancer Staging Manual^31^) in the TCGA cohort. But remained factors such as gender, lymph node metastasis and extrathyroidal invasion and we could not find their association with the expression of SCARA5. In local cohort, we found same trend was consistent with the results of TCGA cohort (Table 2).

**Table 1:** The relationship between SCARA5 and clinicopathologic characteristics in TCGA cohort

Clinicopathologic characteristics	Low expression (%)	High expression (%)	*χ* ^2^	*P*
Age			4.213	.04
≤45	118 (51.1)	96 (41.6)		
>45	113 (48.9)	135 (58.4)		
Gender			2.161	.142
Female	162 (70.1)	176 (76.2)		
Male	69 (29.9)	55 (23.8)		
Tumour size			4.615	.032
≤20 mm	57 (24.7)	78 (33.8)		
>20 mm	174 (75.3)	153 (66.2)		
Extrathyroidal invasion			1.306	.253
Yes	5 (2.2)	2 (0.9)		
No	226 (97.8)	229 (99.1)		
Lymph node metastasis			0.009	.926
Yes	104 (45.0)	105 (45.5)		
No	127 (55.0)	126 (54.5)		
AJCC7			8.657	.003
I + II	167 (72.3)	137 (59.3)		
III + IV	64 (27.7)	94 (40.7)		

Note: *P*‐value <.05.

Abbreviations: AJCC7, Seven Edition of American Joint Committee on Cancer; TCGA, The Cancer Genome Atlas; SCARA5, scavenger receptor class A member 5.

**Table 2:** The relationship between SCARA5 and clinicopathologic characteristics in validated cohort

Clinicopathologic characteristics	Low expression (%)	High expression (%)	*χ* ^2^*	*P*
Age			4.026	.045
≤45	17 (58.6)	9 (32.1)		
>45	12 (41.4)	19 (67.9)		
Gender			0.14	.708
Female	20 (69.0)	18 (64.3)		
Male	9 (31.0)	10 (35.7)		
Tumour size			3.996	.046
≤10 mm	8 (27.6)	15 (53.6)		
>10 mm	21 (72.4)	13 (46.4)		
Unilateral or bilateral			0.16	.689
Unilateral	14 (48.3)	15 (53.6)		
Bilateral	15 (51.7)	13 (46.4)		
Extrathyroidal invasion			0.183	.669
Yes	3 (10.3)	2 (7.1)		
No	26 (89.7)	26 (92.9)		
Lymph node metastasis			0.422	.516
Yes	19 (65.5)	16 (57.1)		
No	10 (34.5)	12 (42.9)		
AJCC7			1.618	.203
I + II	16 (55.2)	20 (71.4)		
III + IV	13 (44.8)	8 (28.6)		

Note: *P*‐value <.05.

Abbreviations: AJCC7, Seven Edition of American Joint Committee on Cancer; SCARA5, scavenger receptor class A member 5.

### 3.3 Downregulated SCARA5 expression was associated with tumour sizes in patients with PTC

Subsequently, we performed logistic regression analysis to investigate whether SCARA5 expression intensified the risk of tumour sizes in PTC patient. In the TCGA cohort, Univariate logistic regression analysis showed that expression of SCARA5 was a significant protective factor for tumour size (odds ratio [OR] = 0.634, 95% confidence interval [CI] = 0.423‐0.951, *P* = .028). We also showed that lymph node metastasis (OR = 2.624, 95% CI = 1.694‐4.066, *P* < .001), Disease stage (OR = 4.372, 95% CI = 2.565‐7.452, *P* < .001) are risk factors for tumour sizes as shown on Table 3.

**Table 3:** Univariate logistic regression analysis for the risk of tumour growth

Factor	OR	95% CI	*P*‐value
SCAR5 expression (high, vs low)	0.634	0.423‐0.951	.028
Lymph node metastasis (N0 vs N1)	2.624	1.694‐4.066	<.001
Age, years (≤45 vs >45)	1.021	0.683‐1.526	.92
Gender (male vs female)	1.578	0.977‐2.550	.062
Disease stage (AJCC7)	4.372	2.565‐7.452	<.001

Note: *P*‐value <.05.

Abbreviations: AJCC7, Seven Edition of American Joint Committee on Cancer; SCARA5, scavenger receptor class A member 5.

Then, the multivariate logistic regression analysis demonstrated that SCARA5 expression (OR = 0.537, 95% CI = 0.340‐0.847, *P* = .008), lymph node metastasis (OR = 1.858, 95% CI = 1.166‐2.959, *P* = .009), disease stage (OR = 4.129, 95% CI = 2.337‐7.292, *P* < .001) intensified the risk of tumour growth (Table 4).

**Table 4:** Multivariate logistic regression analysis for risk of tumour size

Factor	OR	95% CI	*P*‐value
SCAR5 expression (high, vs low)	0.537	0.340‐0.847	.008
Lymph node metastasis (N0 vs N1)	1.858	1.166‐2.959	.009
Disease stage (AJCC7)	4.129	2.337‐7.292	<.001

Note: *P*‐value <.05.

Abbreviations: AJCC7, Seven Edition of American Joint Committee on Cancer; SCARA5, scavenger receptor class A member 5.

### 3.4 Downregulation of SCARA5 promotes cell proliferation in KTC‐1 and BCPAP cell lines

To further investigate the role of SCARA5 in PTC, the expression level of SCARA5 in PTC cell lines (TPC‐1, KTC‐1 and BCPAP) and thyroid normal cell lines (HTORI‐3) were estimated. As shown on Figure 1C, SCARA5 is expressed at higher levels in thyroid normal cell than PTC cell. As a consequence, we chose the relatively higher expression SCARA5 cell lines (KTC‐1 and BCPAP) for our experiment cell lines.

**Figure 2:**
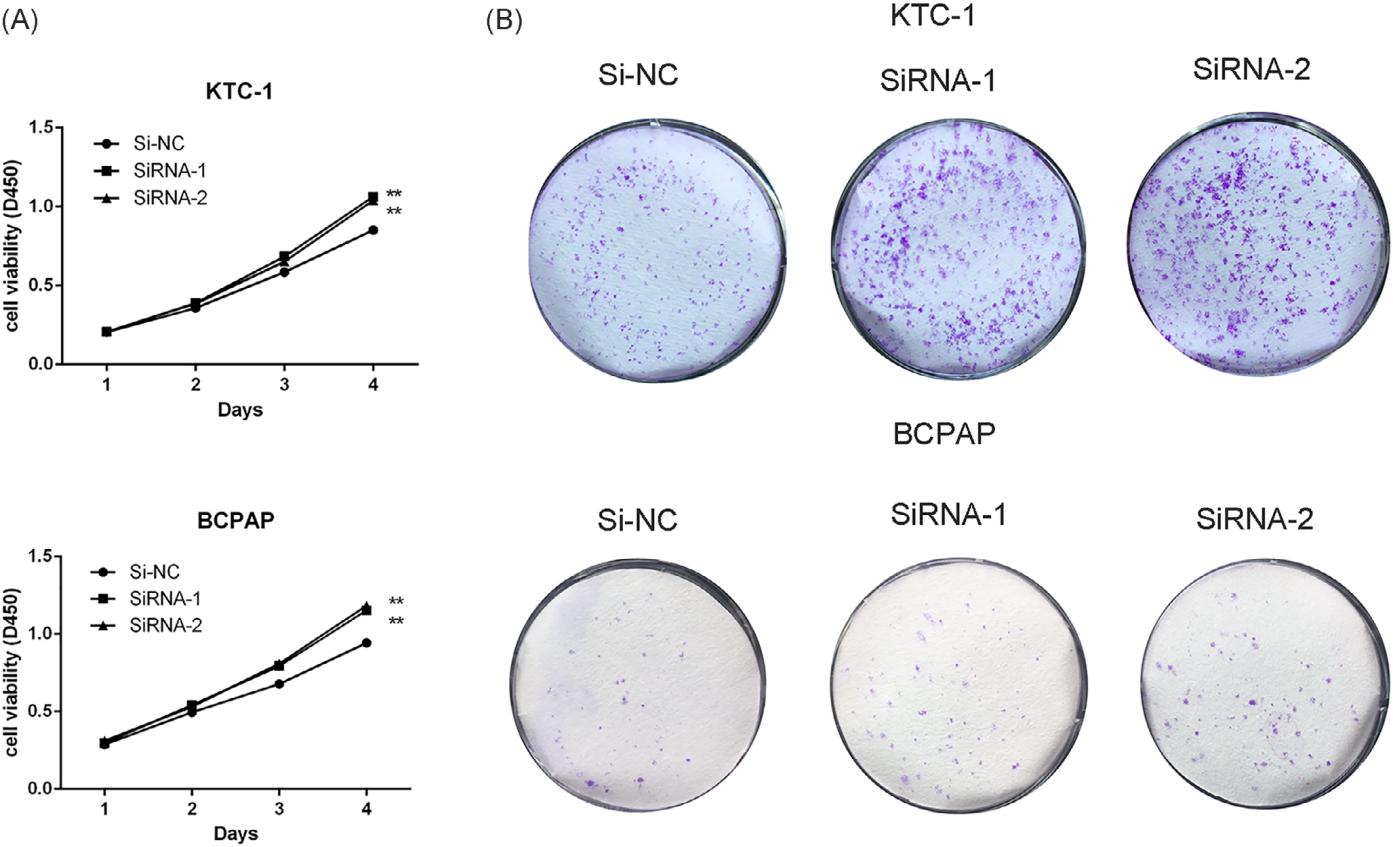
Effect of SCARA5 on proliferation in PTC cell lines. A, KTC‐1 and BCPAP cell lines transfected with Si‐NC, Si‐RNA1 and Si‐RNA2. All cells lines were cultured in 96‐well plates for 1 to 4 days and using CCK‐8 kit to measured cell proliferation. Cell proliferation was significantly promoted in group of Si‐RNA1 and Si‐RNA2. B, Colony formation of Si‐NC, Si‐RNA1 and Si‐RNA2. Colony formation was significantly promoted in group of Si‐RNA1 and Si‐RNA2

Then, two effective Si‐RNA was designed to knockdown the expression of SCARA5 and its interference efficiency was detected by RT‐qPCR (Figure 1D). To manifest if knockdown of SCARA5 promotes the proliferation in KTC‐1 and BCPAP, cell proliferation assay and colony formation assay were performed. As we expected, Downregulation of SCARA5 could promote KTC‐1 and BCPAP cell proliferation (Figure 2A, *P* < .001) and colony formation (Figure 2B) compared with their control group.

### 3.5 Downregulation of SCARA5 promoted the migration and invasion of KTC‐1 and BCPAP cell lines in vitro

**Figure 3:**
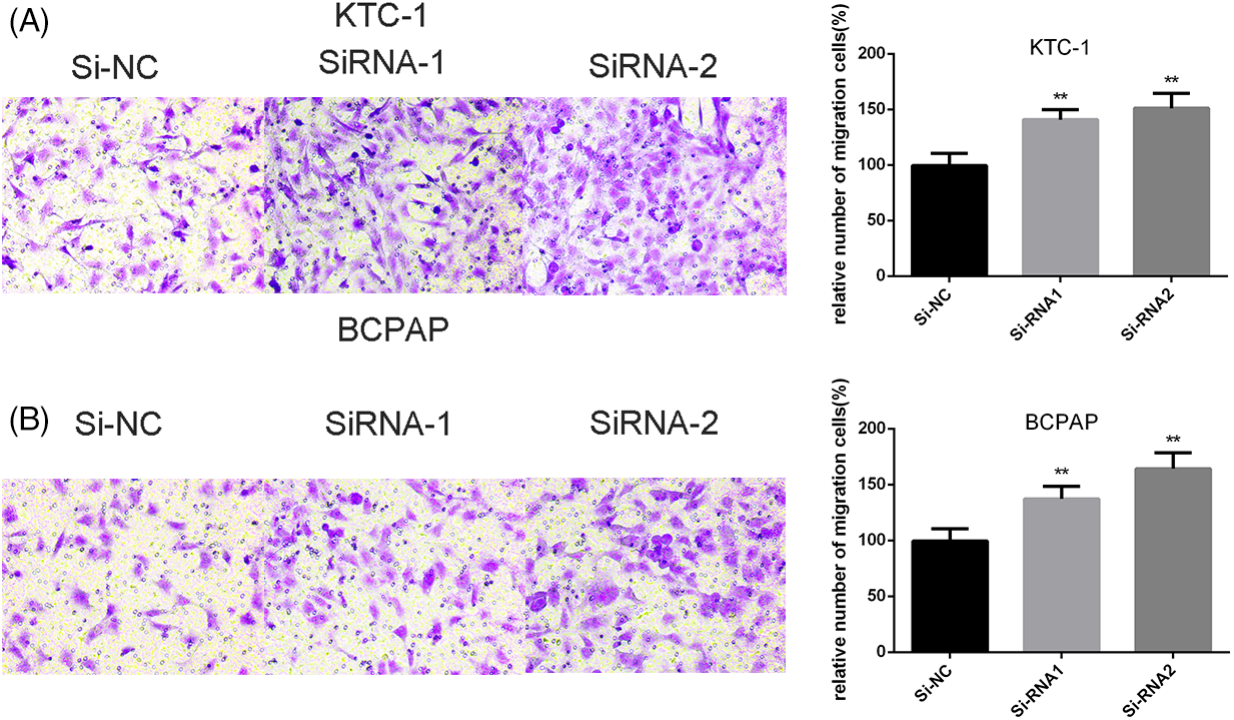
SCARA5 regulates KTC‐1 and BCPAP cell lines migratory capacity in vitro. A‐B, Transwell migration assays in KTC‐1 and BCPAP cells with knockdown of SCARA5 expression and their corresponding control cells. On the right is quantitative results of migration assays. The stained cells were manually counted from five randomly selected fields and normalized with the observed cell proliferation

**Figure 4:**
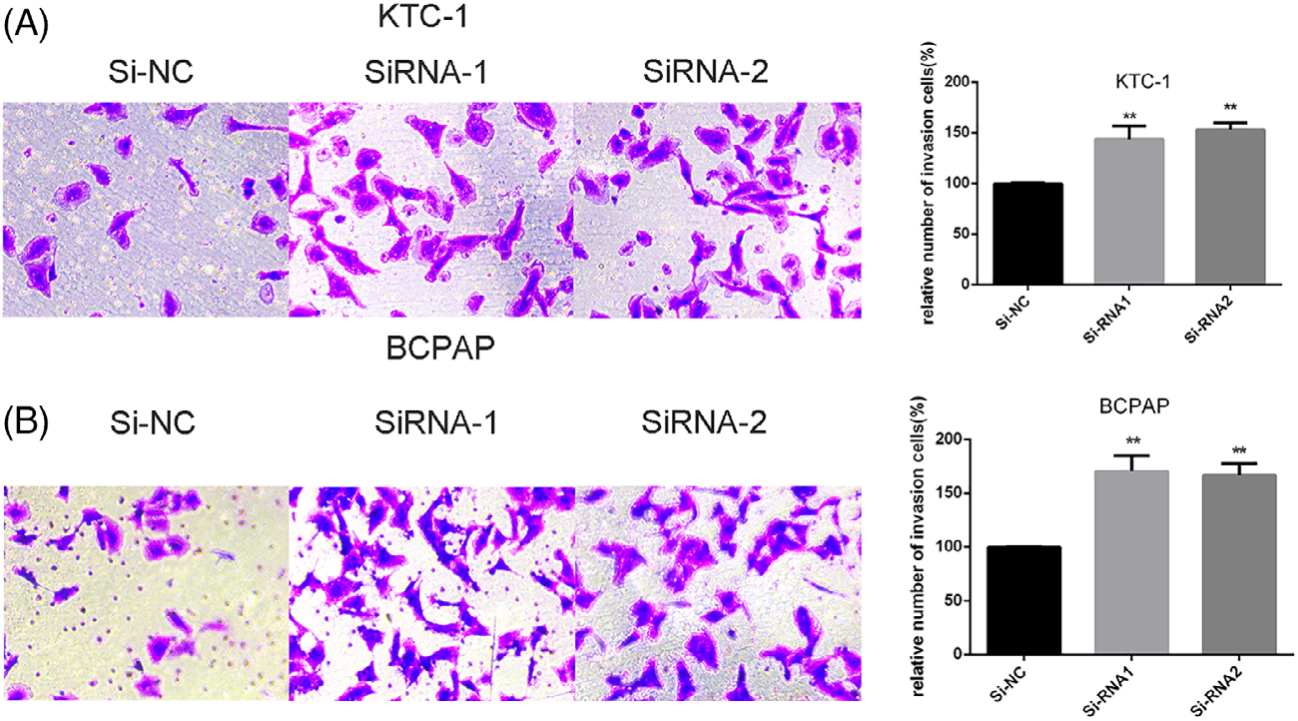
SCARA5 regulates KTC‐1 and BCPAP cell line's ability of invasion in vitro. A‐B, Transwell invasion assays in KTC‐1 and BCPAP cells with downregulation of SCARA5 expression and their corresponding control cells. On the right is quantitative results of migration assays. The stained cells were manually counted from five randomly selected fields and normalized with the observed cell proliferation

We further investigated whether downregulation of SCARA5 could influence abilities of migratory and invasive in KTC‐1 and BCPAP cell lines. The results showed that the migratory ability of Si‐SCARA5 cells increased compared with that of Si‐NC cells (Figure 3, *P* < .001)Besides, results of invasion showed that Si‐SCARA5 cell lines was higher than that of the corresponding Si‐NC controlled cell lines (Figure 4, *P* < .001).

### 3.6 SCARA5 influence in tumorigenesis via modulating epithelial‐mesenchymal transition in KTC‐1 and BCPAP cell lines

**Figure 5:**
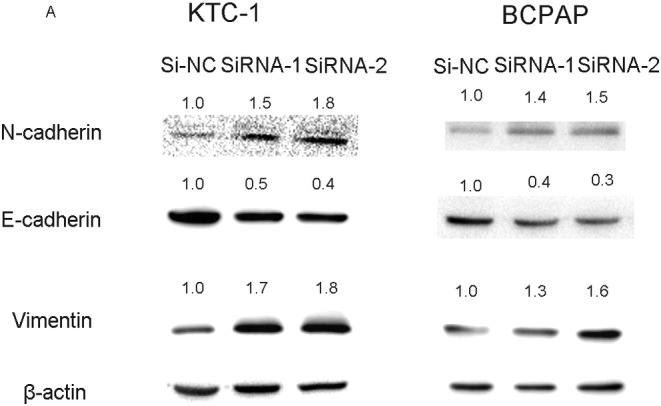
SCARA5 modulates epithelial‐mesenchymal transition (EMT) in KTC‐1 and BCPAP cell lines. Effect of SCARA5 on the related protein of EMT in thyroid carcinoma cell lines (KTC‐1 and BCPAP). The expression level of E‐cadherin N‐cadherin, and vimentin determined by western blotting in transfected KTC‐1 and BCPAP cells

Emerging researches have pointed that epithelial‐mesenchymal transition (EMT) was an indispensable process during tumour progression and metastasis.^32^, ^33^, ^34^ Furthermore, we aimed to found the potential mechanism by which this gene contributes to PTC tumour progression. Though a series of Western blotting, we detected EMT‐related protein expression. As shown in Figure 5, protein expression of E‐cadherin was decreased and Vimentin, N‐cadherin were increased after knocking down SCARA5. We considered that this gene could regulates EMT.

## 4 DISCUSSION

TC is one of the most common endocrine malignancies in the world, and the number of TC cases increases gradually year after year.^35^ A report stated that the number of annual TC cases has increased by 4% globally.^3^ Patience of PTC has a relative good prognosis than other type of cancer,^36^ but it is a nonnegligible problem that lymph node metastasis, capsular invasion and distant metastasis in some PTC patients still occur. A lot of researcher put their effort into reveal molecular mechanisms of TC pathogenesis, but the many unknown oncogenic drivers and epigenetic alterations about this disease are insufficiently known.

With the help of high‐throughput sequencing, our previous study^29^ had found SCARA5 is differentially expressed in 19 pairs of PTC tumours and adjacent normal tissues and might be associated with PTC pathogenesis. This finding indicated that SCARA5 gene likely is an important tumour suppressor in PTC patience.

A member of the scavenger receptor family named scavenger receptor class A member 5 (SCARA5) is located on chromosome 8p21 and its encode protein act as nontransferrin iron delivery receptor to bound iron in normal physiological conditions. Recently, more and more studies reported this gene act as a suppressor gene and down‐expression of SCARA5 was correlated with several malignancies. Huang et al discovered that suppression of SCARA5 was also correlates with cellular invasion and overall progression in HCC.^26^ Furthermore, Xin‐Zhu Wen et al demonstrated that overexpression of SCARA5 could inhibit osteosarcoma proliferation and invasion though FAK signalling pathway.^37^ However, the function of SCARA5 in TC still remain to be fully elucidated.

In this study, we examined 57 matched PTC tumour tissue and adjacent normal tissues to testified the function of SCARA5 in PTC. The mRNA expression level of SCARA5 was evaluated in local cohort and different PTC cell lines via RT‐qPCR. in vitro experiments illustrated that downregulation of SCARA5 induced PTC cell proliferation and the abilities of migration and invasion. Finally, we measured the protein expression of EMT‐related molecules via immunoblotting and found low E‐cadherin and high N‐cadherin and vimentins expression in the Si‐SCARA5 cell lines.

There are some limitations still exist in current study. First, the relationship between SCARA5 and the prognosis of PTC in large samples needs to be investigated. Second, the exact molecules involved in the role of SCARA5 to modulate EMT pathway in PTC tumorigenesis need further study. In addition, animal experiments should be performed to verify the anti‐tumour function of SCARA5.

In a summary, the present study investigated the association between SCARA5 and tumour growth in TCGA cohort and local cohort. And, downregulation of SCARA5 induce TC cell proliferation, colony formation, migration, and invasion though EMT pathway. These fascinating findings provide potential molecular markers for the diagnosis and treatment of PTC.

## CONFLICT OF INTEREST STATEMENT

The authors declare no conflicts of interest.

## Supporting information


**Data S1**. Supporting Information

## Data Availability

The datasets supporting the conclusions of this study and additional images are included in this article. Raw data are available on the main electronic data storage system of The First Affiliated Hospital of Wenzhou Medical University, and access can be provided upon request to the authors.
