# Supplementary material for: Scavenger receptor class A, member 5 is associated with thyroid cancer cell lines progression via epithelial‐mesenchymal transition
Source: Cell Biochem Funct. 2020 Jan 27;38(2):158–66. doi: 10.1002/cbf.3455 (PMC7216911; doi:10.1002/cbf.3455)
Supplement: Supplementary file 1 — Data S1. Supporting Information [file CBF-38-158-s001.docx]

**Response to Reviewer**

Dear Prof. Zhijie Chang 

It is with excitement that I resubmit to you a revised version of manuscript. **Manuscript ID CBF-19-0220**, Title: **Scavenger receptor class A, member 5 (SCARA5) is associated with thyroid cancer cell lines progression via epithelial-mesenchymal transition.**

Thank you for giving me the opportunity to revise and resubmit this manuscript. In keeping with my last communication with you, I am resubmitting this revision before the agreed upon deadline. I appreciate the time and details provided by each reviewer and by you and have incorporated the suggested changes into the manuscript to the best of my ability. The manuscript has certainly benefited from these insightful revision suggestions. I look forward to working with you and the reviewers to move this manuscript closer to publication in the **Cell Biochemistry & Function.**

Yours sincerely

Dr. Zheng

----Reviewer(s)' Comments to Author: -----

PEER REVIEW INFORMATION

Referee(s)' Comments to Author:

Reviewing: 1

Comments to the Author

In this manuscript, the authors showed that Scavenger receptor class A, member 5 (SCARA5) was associated with thyroid cancer progression via regulating the epithelial-mesenchymal transition. The data are generally supportive of the conclusions. However, the data must be strengthened in a number of areas.

1, the English is poor, like “we were collected 57 paired of primary PTC tissue” and “we were also estimated the expression level”. Please reorganize it, and also the words size.

Thanks for your carefulness. In our manuscript, there are some mistakes and errors. We have tried our best to revise and correct the mistakes and have tried to present our best version of manuscript.

2, in Fig 1D, please also show the protein level to confirm the knockdown of SCARA5.

This is a good point. We have performed western blotting of SCARA5 in KTC-1 and BCPAP cell lines. The results revised in Figure 1E as follow:

3, for the phenotypes induced by SCARA5 knockdown, it’s better to use shRNA instead of siRNA, as siRNA can only work for about 3 days.

Thanks for your thoughtful suggestion. In this experiments, we used modified siRNAs to knockout SCARA5. Chiu YL et al proved that persistence of modified siRNAs interference in vivo can last 7-10 days (DOI: 10.1261/rna.5103703) . Therefore, we considered it adequate for our experiment purpose.

4, it will make the conclusion more solid if the authors could prove that the overexpression of SCARA5 could rescue the phenotypes induced by SCARA5 knockdown.

Thanks for your carefulness. We have tried to overexpress SCARA5 in KTC-1 and BCPAP cell lines , however the background of those cells are too high to overexpress.

5, Is there any explanation for how SCARA5 affected the expression of EMT marker?

We've been thinking about this problem. There are many pathways could influence EMT marker in thyroid cancer. This is where we need to explore. We still will continue more experiment in the future. But in this time, we have many limitations in our experiment such as limited amount of fund and manpower.
